# Characterization of Airborne Particles Emitted During Application of Cosmetic Talc Products

**DOI:** 10.3390/ijerph16203830

**Published:** 2019-10-11

**Authors:** Pat E. Rasmussen, Christine Levesque, Jianjun Niu, Howard D. Gardner, Gregory Nilsson, Kristin Macey

**Affiliations:** 1 Environmental Health Science and Research Bureau, HECSB, Health Canada, 50 Colombine Drive, Ottawa, ON K1A 0K9, Canada; christine.levesque@canada.ca (C.L.); jianjun.niu@canada.ca (J.N.); 2 Earth and Environmental Sciences Department, University of Ottawa, Ottawa, ON K1N 6N5, Canada; dave.gardner@uottawa.ca; 3 Construction Research Center, National Research Council of Canada, 1200 Montreal Road, Ottawa, ON K1A 0R6, Canada; Gregory.Nilsson@nrc-cnrc.gc.ca; 4 Existing Substances Risk Assessment Bureau, HECSB, Health Canada, 269 Laurier Ave W., Ottawa, Ottawa, ON K1A 0P8, Canada; kristin.macey@canada.ca

**Keywords:** inhalation exposure, talcum powder, magnesium silicate, particulate matter, consumer products, short-term variability

## Abstract

A pilot study was undertaken to characterize the concentration, duration and particle size distribution of the talc cloud that forms in the personal breathing zone (PBZ) during application of certain talc-containing cosmetics. Multiple direct-reading instruments were employed to simultaneously monitor PM_4_ concentrations (particulate matter with aerodynamic diameter < 4 µm; mg/m^3^) at different distances from each of three subjects while they applied talc products. Results indicated that the purpose and method of applying the talc product, combined with behavioral and physical differences amongst subjects, all strongly influenced airborne talc concentrations and the duration of the cloud. Air concentrations of talc in the PBZ averaged around 1.0 mg/m^3^, and the duration of exposure varied from less than one minute to more than ten minutes. The real-time monitors captured the occasional formation of secondary clouds, likely caused by resuspension of talc particles from skin or other surfaces. Measurements of aerosolized baby powder, face powder, and two adult body powders indicated that the median aerodynamic diameter of the talc cloud ranged from 1.7 to 2.0 µm. These direct-reading approaches were valuable for providing detailed characterization of short duration exposures to airborne talc particles, and will be useful to support future exposure assessments of talc and other powders in consumer products.

## 1. Introduction

Powdered talc (hydrous magnesium silicate) is commonly used as the principal ingredient in baby, body and face powder products. Talc used in cosmetics (cosmetic grade talc) should be free of asbestos and not contain any detectable fibrous amphibole or free crystalline silica [1,2]. Nevertheless, concern remains about the potential risk of inhaling insoluble respirable talc particles during the application of talc-containing cosmetics, based on evidence of non-neoplastic pulmonary lesions found in studies conducted with rats and mice chronically exposed to cosmetic grade non-asbestiform talc [3].

Several studies over the past four decades have used a variety of approaches to quantify air concentrations of talc particles during the use of cosmetic talc products. Aylott et al. [4] measured the <7 µm fraction of airborne particulate matter (PM) generated during the use of loose face powder, adult dusting powder, baby dusting powder and micronized adult dusting powder, based on atomic absorption analysis of Mg on membrane filter samples after 5 min collection (flow rate 1.9 L/min). Russell et al. [5] simulated exposures of babies to talcum powder during diapering and of adults while applying talcum powder to themselves, and measured airborne talc concentrations (<10 µm) using a cyclone sampler and quartz crystal mass monitor (flow rate 1.0 L/min). Recently, Anderson et al. [6] conducted an exposure study designed to quantify respirable talc concentrations in the breathing zone, in which five subjects applied historical talcum powder products (purchased in the 1960s and 1970s) while wearing personal filter-based samplers. To ensure that sufficient sample mass was collected on the filters for quantification by gravimetric analysis, each exposure simulation consisted of eight application events at six-minute intervals for a total sampling duration of 48 min [6].

While these studies provide evidence that inhalation exposures to airborne talc particles occur during application of talc-containing cosmetics (e.g., baby, body, and face powders), certain data gaps and uncertainties should be addressed to better characterize the talc cloud created during the use of these products. One data gap is the particle size distribution of airborne talc particles. Aylott et al. [4], Russell et al. [5] and Anderson et al. [6] quantified airborne PM_10_ (particles with median aerodynamic diameter (MAD) < 10 µm; also called the “thoracic fraction”) or PM_4_ (particles with MAD < 4 µm; also called the “respirable fraction”) during the use of talcum powders. Using scanning electron microscopy (SEM) and energy-dispersive X-ray analysis (EDX), Conner et al. [7] identified the presence of talc particles in indoor residential PM_2.5_ (particles with MAD < 2.5 µm; also called the “fine particle fraction”). They concluded that fine talc particles are likely to be present at high concentrations for short time periods (corresponding to the time required for application) in indoor microenvironments, and should be considered as possible sources of fine particle exposure [7]. However, subsequent particle size distribution measurements [8] indicated a mean diameter of 14.3 µm for talcum powder particles, that is, much larger than the PM_2.5_ talc particles observed by Conner et al. [7]. These somewhat contradictory findings indicate the need to clarify the particle size distribution of commonly used talc products. Another source of uncertainty is the analytical challenge posed by the short duration and low concentration of particles produced during a single application of talcum powder [5]. For example, the above-mentioned 48 min measurement window of the Anderson et al. study [6] was needed to accumulate enough particle mass on the sample filters to exceed the detection limit associated with gravimetric analysis. However, most other studies report that the typical duration of talc exposure is between 0.5 and 5 min [9,10] and, thus, the influence of any “dead time” between application events within the 48 min time-weighted average reported by Anderson et al. [6] is an unknown. (In this context, “dead time” refers to the interval between applications in which airborne talc particles have settled and air concentrations have returned to background levels.)

The present pilot study used real-time, direct-reading instruments to capture short-term temporal variations in respirable particle concentrations (PM_4_ in mg/m^3^) during talc product application, with the goal of collecting information at a level of detail that would assist in the design of future exposure studies. While the quartz crystal technology employed by Russell et al. [5] was a prototype of modern particle monitors, the real-time personal samplers and area monitors used in the present study were more sensitive and had a faster response time than previously available technologies. These monitors permitted simultaneous measurements of respirable particle concentrations at three distances from the face (0, 30 and 53 cm) for each of three subjects while they applied talc products. By determining the particle size distribution of cosmetic talc products in current use, and by characterizing the duration and concentration of the talc cloud that forms during real-life application conditions, this pilot study provides additional information for the assessment of risk associated with inhalation exposures of powder-based cosmetics.

## 2. Materials and Methods

### 2.1. Selection and Characterization of Talcum Powder Products

Four cosmetic products commercially available in Canada (one face powder, one baby powder and two adult body powders) that contain talc (Mg_3_Si_4_O_10_(OH)_2_; CAS RN 14807-96-6) as the major component were selected for use in laboratory chamber experiments and exposure experiments using human subjects. An Ultima IV X-ray diffractometer (XRD; Rigaku Americas Corp., The Woodlands, TX, USA) with integrated X-ray powder diffraction software (PDXL version 2.8.1.1; Rigaku Americas Corp., The Woodlands, TX, USA) and the 2016 version of PDF-2 application database software (ICDD -International Centre for Diffraction Data, Newtown Square, PA, USA) were used to process diffraction patterns and provide a list of identified substances in each product (limit of detection approximately 1%). The instrument was configured to use parafocusing Bragg–Brentano geometry, using a Cu target with an acceleration voltage of 40 kV and a tube current of 44 mA, as indicated in Table 1. Prior to the XRD measurements, the organic component of the talc products was removed using 3% hydrogen peroxide, and the weight of the organic fraction was determined by subtraction.

### 2.2. Instruments Used for Monitoring Airborne Talc

Particle size distributions of aerosolized talc particles were measured using an Aerodynamic Particle Sizer (APS; TSI Inc. Model 3321; MN, USA). A Scanning Mobility Particle Sizer (SMPS; TSI Inc. Model 3788/3082; MN, USA) was used to characterize the particle size distribution in the submicron (<1 µm) range. Two DustTrak DRX Aerosol Monitors (TSI Inc. Model 8533; MN, USA) were used for area measurements of size segregated aerosol mass concentration (i.e., PM_10_, PM_4_, PM_2.5_, and PM_1_). The DRX, which combines photometry and single particle sizing in one optical device, has the capacity to measure aerosol mass concentration in real time (recorded each sec) over a wide concentration range (0.001–150 mg/m^3^) [11]. Where appropriate, additional area measurements (PM_10_ only) were made using a DustTrak II (TSI Inc. Model 8530 DustTrak with PM_10_ impactor; MN, USA). Prior to the experiments, the DRX and DustTrak II instruments were calibrated by the manufacturer using Arizona Test Dust (ATD; ISO 12103-1, A1 dust). Calibration using ATD is appropriate for measuring airborne talc particle concentrations due to the similar specific gravity of ATD (2.5–2.7 g/cm³) compared to talcum powder (2.6 to 2.9 g/cm³). Personal breathing zone (PBZ) measurements of respirable (PM_4_) talc concentrations were made in real-time using DustCount monitors (Model 8899; 5 s logging period; Nanozen Industries Inc., BC, Canada). The DustCount is a direct-reading personal particle monitor with a PM_4_ impactor as defined by NIOSH [12]. The unit includes single particle size selection and mass conversion using 20 particle size bins (0.5 µm to 10 µm) and has a detection range of 1.00 µg/m^3^ to 21.50 mg/m^3^. Although both the DRX and DustCount monitors permit the collection of a simultaneous filter sample, efforts to collect a filter-based sample in the present study were unsuccessful. The small particle masses that accumulated on the sample filters over the short duration of the talc clouds were inadequate to permit accurate and reproducible gravimetric measurements. 

### 2.3. Measurements in a Controlled Laboratory Environment

A controlled laboratory study was undertaken using a baby powder product to simulate the study design of Anderson et al. [6], which consisted of eight sequential application events at six minutes intervals over a 48 min period. In the Anderson et al. study [6], the duration of an “application event” ranged from 13 to 47 s and included the actions of shaking the bottle and rubbing the talc product on the body [6]. The number of shakes in the Anderson et al. study ranged from approximately three (Subjects 1 and 4) to more than 20 (Subjects 3 and 5) for a single application event, with the average mass of powder per application ranging from 0.7 to 3.4 g. To mimic these conditions as much as possible in the present chamber study, each application event consisted of three squeezes of the bottle within a 15–40 s period to release the powder (through holes in the bottle top) and generate a talc particle cloud. The average mass of baby powder released per squeeze in the present study was 0.23 ± 0.14 g (based on 6 replicate measurements), resulting in an average mass of powder per application of 0.7 g (i.e., for three squeezes). The simulation was conducted in triplicate inside a custom 168 by 53 cm self-ventilating polypropylene fumehood (Design Filtration Inc., Ottawa, Canada), with the intake of the DRX DustTrak monitor positioned at 53 cm from the bottle top. The fumehood was cleaned and certified (with replacement of High Efficiency Particulate Arrestance (HEPA) filters) prior to the experiments and was cleaned and vacuumed between experiments. During the aerosolization experiments, the fumehood functioned as a laboratory chamber (air volume 0.77 m^3^), with the panel doors closed (as far as possible) and the fan turned off so that the air exchange rate was effectively zero, to be consistent with the zero exchange rate used by Anderson et al. [6] and earlier studies. 

Additional measurements were conducted inside the chamber to compare airborne concentrations created during the use of three different products: Two body powders and one baby powder product. All products were dispensed from their original product containers as purchased, through holes in the bottle tops. Three direct reading optical monitors (DustTrak) were used to measure the duration and concentration of the particle cloud formed from a single squeeze of the container. DustTrak measurements were made simultaneously at three distances from the bottle top (DustTrak II at 30 cm, DRX at 53 cm, and DRX at 69 cm), that were being considered for the exposure study. Each set of measurements was conducted in triplicate to report mean ± SD for each product. Temperature and humidity inside the chamber were monitored throughout the experiments.

### 2.4. Measurements in a Residential Bathroom During Application of Talc Products by Human Subjects

A detailed pilot exposure study was conducted using human subjects to obtain information that would assist in the design of future studies of exposure to talc-containing consumer products. This study received approval on 14 May 2018 from the Health Canada and Public Health Agency of Canada Ethics Review Board (Project ID 2017-0042), and informed consent was obtained from all volunteer subjects. The objective was to characterize air concentrations of talc particles using real-time monitors, while male and female adult volunteer subjects applied either a baby powder or a face powder to themselves following their own routine method of application. Continuous, direct-reading measurements of PM_4_ airborne particle concentrations (mg/m^3^) were made as subjects applied talc powder while sitting on a stool in a residential bathroom (air volume 9.5 m^3^). Observations about the behavior of each participant (e.g., amount of head movement, body movements, application timing and style) were recorded during each application. During the study, all participants were provided with a half face mask respiratory assembly (North Silicone Half Mask 7700 Series with a 7583P100 North filter cartridge) in order to mitigate any potential inhalation exposure.

PM_4_ concentrations were monitored at three distances from the face (0, 30 and 53 cm) during product application. Each subject wore a DustCount personal monitor (5 s logging period) on their waist (attached to a belt), and the unit was equipped with flexible graphite-impregnated silicon tubing to position the air intake between the subject’s nose and ear (i.e., distance of 0 cm from the subject’s face). The intake for a DustTrak DRX monitor (1 s logging period) was affixed in a stationary position 1.1 m above the floor and within 30 cm of the subject’s face. To obtain simultaneous stationary area measurements, intakes for another DustCount unit and another DustTrak DRX were positioned side-by-side 45 cm above the floor and approximately 53 cm from the subject’s face. There was no significant difference in results from the collocated DustCount and DRX units (averaged over 5 s intervals for both models) whether evaluated using a Student’s t-test (*p* = 0.18) or Wilcoxon signed rank test (*p* = 0.36). Thus, DustTrak DRX results (1 s logging) are reported herein to represent the 53 cm area measurements.

The bathroom door and window were closed during applications and the bathroom fan was turned off. Temperature and humidity in the bathroom were recorded throughout the experiments. All applications were run in triplicate to determine mean and standard deviation (mean ± SD) air concentrations over time, and the floor was cleaned between each application to minimize contributions from dust resuspension.

## 3. Results

The physical and chemical characteristics of four cosmetic talc products used in the present study are presented in Table 2. Measurements of airborne particle size distribution for the talc products used in the present study indicated that median aerodynamic diameter ranged from 1.7 to 2.0 µm (i.e., within the PM_2.5_ or fine particle range) depending on the product (Table 2). Particle size distributions (#/cm^3^) for the four studied talc products (presented in the Supplemental Information; Figures S1 and S2) ranged from <1 µm to 8 µm in particle size. Sporadic peaks were identified in the submicron range of the baby powder and two adult body powders (peak modes within the 141 to 289 nm range determined using SMPS; Figure S1). All products displayed a predominant peak in the PM_2.5_ range (peak modes within 1.6 to 2.1 µm determined using APS; Figure S2).

### 3.1. Laboratory Chamber Simulation

**Figure 1:**
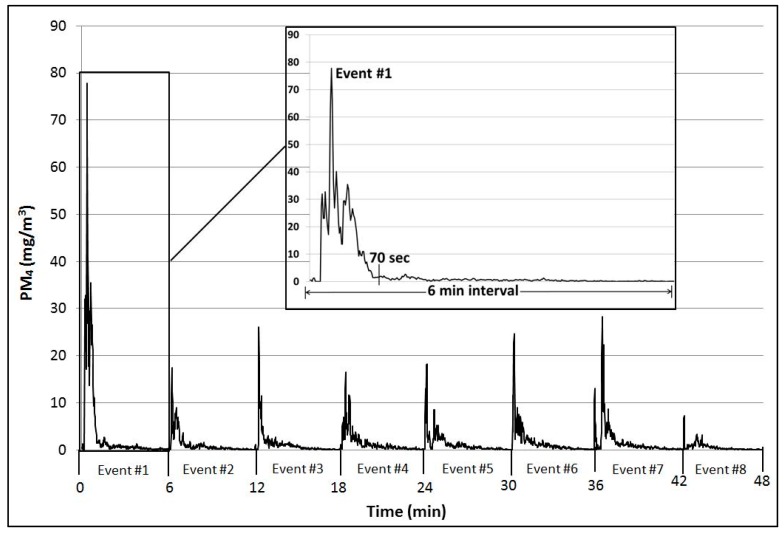
Respirable talc concentrations (PM_4_) monitored in a laboratory chamber (temp. 26.5–27.0 °C and RH 20–21.2%) during eight application events (at 6 min intervals) over 48 min. Each application event consisted of three squeezes of a talc product bottle (baby powder). Inset shows details of Event 1.

The primary purpose of conducting measurements under controlled laboratory conditions was to enable a comparison of results generated by real-time (direct-reading) instruments used in the present study with the Anderson et al. [6] time-weighted averages obtained using filter-based samplers and gravimetric analysis. The real-time PM_4_ measurements for the simulation run of 48 min in the present study, with eight application events at six-min intervals, are shown in Figure 1. The weight of talc released in each “three-squeeze” application event in the present study (average 0.7 g) fell at the low end of the range of 0.7–3.4 g per application event reported by Anderson et al. [6]. The average PM_4_ concentration over 48 min in the present study was 1.58 mg/m^3^, which fell within the range of 0.26 to 5.03 mg/m^3^ respirable talc reported by Anderson et al. [6] for five subjects monitored over 48 min and was similar to their reported overall average of 1.46 mg/m^3^. Thus, despite differences in measurement technologies and experimental conditions, the 48 min average talc exposure reported by Anderson et al. [6] was comparable to that observed in the present study.

However, if the eight primary cloud events in Figure 1 are considered individually, then the average PM_4_ concentration for Figure 1 increases to 5.13 ± 4.90 mg/m^3^ (mean ± SD) for an average talc cloud duration of 1.62 ± 0.5 min. The reason for the difference is the average “dead time” of 4.38 ± 0.5 min between applications (when the PM_4_ concentration in Figure 1 approaches background) that is included in the 48 min average. For example, the PM_4_ concentration is 16.89 mg/m^3^ over the 70 s duration of the talc cloud in Event 1 (Figure 1 inset), but the PM_4_ concentration decreases to 3.77 mg/m^3^ when averaged over the entire 6 min interval. The results in Figure 1 highlight the impact of the time period considered—that is, averaging measurements over a longer time period than the actual duration of the talc cloud will result in an underestimate of airborne talc concentrations. Thus, the advantage of using real-time direct-reading monitors, compared to gravimetric approaches, is the more accurate interpretation of the airborne talc concentration over the short duration of the particle cloud.

### 3.2. Comparison of Products under Controlled Chamber Conditions

The second purpose of the chamber study was to compare three commercially available talc powder products (1 baby and 2 body powders) with respect to the duration and concentration of inhalable talc (PM_10_) in the particle cloud that forms during application under controlled laboratory conditions. A single squeeze of the container was used for this comparison study. Measurements taken at three distances are summarized in Table 3. Overall, the duration of the particle cloud and concentration of PM_10_ in the particle cloud was 463 ± 133 s (7.7 ± 2.2 min) and 2.76 ± 2.11 mg/m^3^ respectively (based on mean ± SD of last line in Table 3; i.e., three products at three distances).

Differences amongst the time and duration of the particle clouds formed by the three products (Table 3) may be related to differences in the number and type of ingredients. Body powder #1, which contained the greatest number of ingredients (listed in Table 2), also displayed the longest duration (509 ± 99 s) and the highest air concentration (5.02 ± 3.170 mg/m^3^). It was also noted that the weight of body powder #1 released per squeeze averaged 0.33 ± 0.34 g compared to the baby powder (0.23 ± 0.14 g per squeeze) and body powder #2 (0.28 ± 0.44 g per squeeze). Although Aylott et al. [4] found no evidence that the presence of perfume in talc products affected airborne concentrations, these results suggest that other ingredients may have an effect. 

The comparison of products in Table 3 is based on measurements of PM_10_ rather than PM_4_ because only two DRX units were available (employed at 53 cm and 69 cm), requiring the use of a DustTrak II unit (equipped with a PM_10_ impactor) for the 30 cm measurement. Wang et al. [11] reported that DustTrak II results may not be directly comparable with DRX results, as the former relies on a photometric signal which tends to underestimate the mass concentration of coarse particles (in contrast to the DRX which uses optical counting to measure coarse particles and a photometric signal for the fine fraction). Therefore, while Table 3 is useful for comparing products, it is not useful for assessing concentration vs. distance as the higher concentrations at 53 cm (compared to 30 cm) are influenced by the difference between the DRX and DustTrak II technologies.

### 3.3. Exposure Measurements Using Human Subjects

In order to inform the design of future exposure studies, this human exposure pilot study aimed to measure air concentrations of talc particles using real-time monitors during “typical” applications of talc-containing cosmetic products in a bathroom setting, using three adult volunteers. Subject A was a female who applied baby powder (Table 2; row 1) to her upper torso and arms according to her normal morning routine. Subject B was a female who applied the cosmetic face powder (Table 2; row 4) according to instructions provided by the manufacturer, which involved using the supplied powder puff to apply the product to the face, and then brushing off the excess powder after a 2 min “setting” period. Subject C was a male who used baby powder (Table 2; row 1) to assist in putting on a tight-fitting “shorty” style wetsuit as he normally would for water sports (e.g., scuba diving, surfing, sailing). 

**Figure 2:**
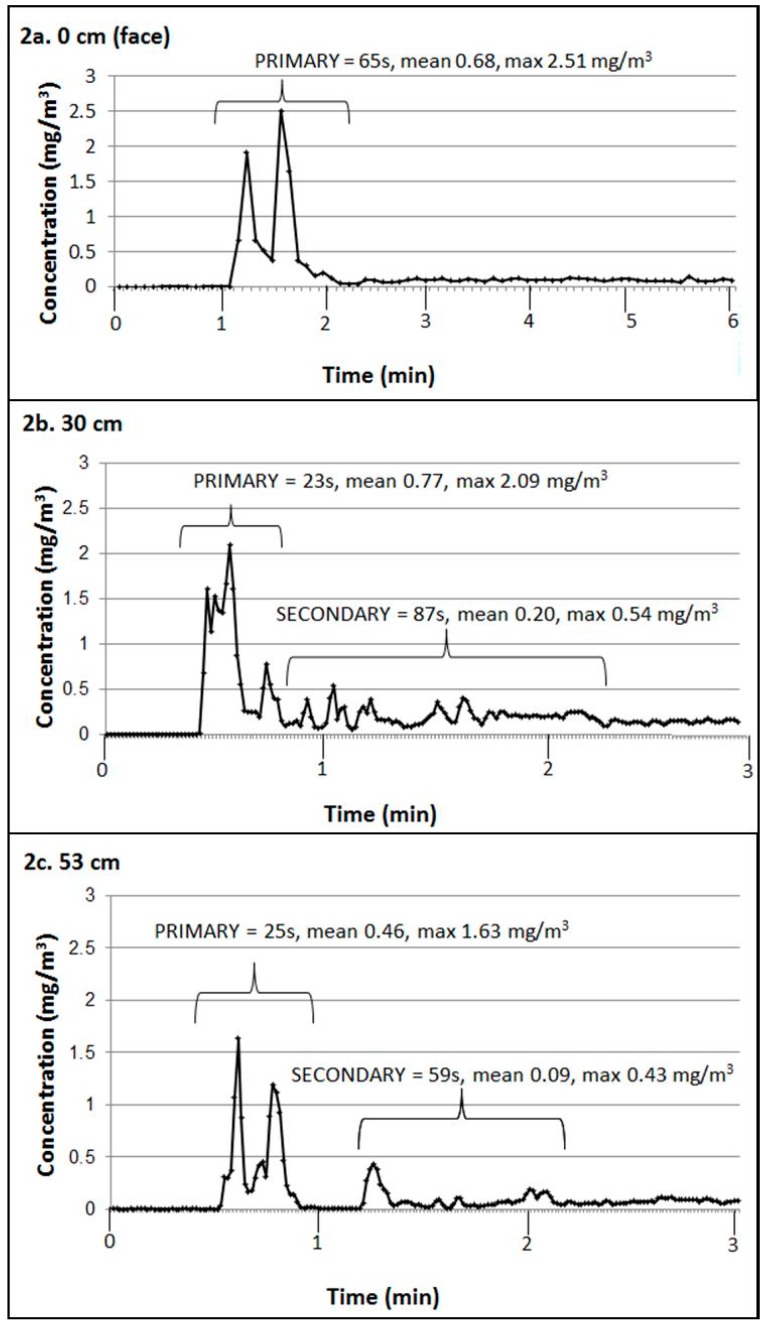
Measurements of PM_4_ concentration and duration of primary and secondary talc clouds formed when Subject A applied talcum powder. Simultaneous measurements were taken in the personal breathing zone at the face (**a**) and 30 cm from the face (**b**), plus an area measurement taken 53 cm from the face (**c**).

Figure 2 provides examples of the variation in particle concentration (mg/m^3^; y-axis) over time (*x*-axis), during talc product application by Subject A. Two sets of measurements were made within the PBZ at 0 cm (Figure 2a) and 30 cm (Figure 2b); while the third was an area measurement at 53 cm from the subject’s face (Figure 2c). 

In most, but not all, applications, formation of a secondary talc cloud was observed, defined as measurements exceeding noise (calculated as 10 times the SD of background) for a minimum duration of 30 s. In some situations, the secondary cloud was continuous with the primary cloud (i.e., a “shoulder” as the primary cloud dissipated), as shown in Figure 2b. In other situations, the secondary cloud occurred as a distinct event separated by time, as in the area measurement shown in Figure 2c.

Table 4 summarizes the concentrations and durations of talc clouds observed for all three subjects (mean ± SD for *n* = 3 replicates per subject). The direct-reading DustCount monitor (positioned at 0 cm, beside the nose) measured a mean ± SD PM_4_ concentration of 0.48 ± 0.18 mg/m^3^ for the primary cloud that formed when Subject A applied baby powder. Average PM_4_ concentrations of 1.80 ± 0.82 mg/m^3^ were measured for Subject B applying loose face powder, and 0.61 ± 0.09 mg/m^3^ for Subject C applying baby powder, combining primary and secondary talc clouds in both cases (Table 3). The overall mean ± SD PM_4_ concentration of airborne talc was 0.96 ± 0.73 mg/m^3^ for the three subjects in Table 4 (for distance = 0 cm, combining primary and secondary clouds). These concentrations observed within the PBZ fell within the range of concentrations measured for five subjects by Anderson et al. [6]. 

Measured PM_4_ concentrations were generally lower at a distance of 53 cm than within the PBZ (Table 4). The application of loose face powder with a puff applicator resulted in the highest average PM_4_ concentration in the immediate vicinity of the nose (0 cm). Table 4 shows that the concentration of the primary cloud was approximately four-fold higher at 0 cm than at 30 cm (2.48 mg/m^3^ compared to 0.60 mg/m^3^ respectively) in the case of face powder application. 

Average PM_4_ concentrations of the primary cloud for Subject A and Subject C, who both applied baby powder to their bodies, were notably similar at 30 cm (1.10 mg/m^3^ and 1.00 mg/m^3^, respectively). The key difference between Subjects A and C at 30 cm was the longer duration of Subject C’s talc cloud (386 ± 93 s compared to 73 ± 46 s), caused by Subject C taking a longer time to apply the product over a larger skin area and to apply the product to the inside of his wetsuit. Another difference was related to greater movement of Subject C’s head during the application, compared to Subject A who remained quite stationary, and generally kept her head above the main zone of application. More head movement resulted in a more homogeneous distribution of talc particles within the PBZ for Subject C (0.93 mg/m^3^ at the face and 1.00 mg/m^3^ at 30 cm) than for Subject A (0.48 mg/m^3^ at the face and 1.10 mg/m^3^ at 30 cm; Table 4 primary cloud). 

## 4. Discussion

The use of real-time direct-reading monitors in the present study permitted investigation of the shape of the talc cloud that forms in the PBZ during product application (i.e., changes in airborne particle concentrations over a short time). Measurements of particle size distribution (Table 2 and Figures S1 and S2) indicated that while all of the particles in the airborne talc cloud were within the thoracic size fraction of inhalable particles (PM_10_), the majority of the particles were within the respirable size fraction (PM_4_); in fact, the median values of all studied products fell within the subset of particles known as the fine fraction (PM_2.5_). Results from the pilot exposure study, which were intended to assist in the design of future exposure studies, showed that airborne talc clouds that form during application of talc-containing products by test subjects are characterized by high variability—in terms of shape, concentration and duration. An interesting feature was the occasional formation of secondary clouds, most likely caused by resuspension of talc particles from skin or other surfaces.

The pilot exposure study also demonstrated the variability of talc cloud characteristics. These observed differences may be due to proximity of the sampler intake to the area of talc application, the purpose of the talc application, and differences in behavior amongst subjects (Figure 2 and Table 4). The detailed measurements at different distances in the present study will help inform future study designs, as the results showed opposite trends in concentrations within the PBZ for different application scenarios. During application of face powder, the concentration of the primary cloud was approximately four-fold higher at the subject’s face (0 cm) compared to 30 cm from the face (2.48 mg/m^3^ vs. 0.60 mg/m^3^, respectively). The reverse trend (i.e., lower concentration at 0 cm than at 30 cm) was observed during application of talc to the body by the adult female (0.48 mg/m^3^ vs. 1.10 mg/m^3^, respectively). Thus, to capture the correct exposure concentration, the purpose and method of product application must be taken into consideration for selection of the optimal placement of the sampler intake.

### 4.1. Variability of Exposures Amongst Subjects

The high variability of exposures amongst different subjects applying various talc-containing products observed in the current study has been noted by previous researchers. For example, Russell et al. [5], who used personal monitors based on early direct-reading technology, reported that the average respirable talc concentration observed for adult exposure (2.03 mg/m^3^) was over ten times greater than that found for infants (0.19 mg/m^3^). Adults were also exposed for a longer time period—1.23 min compared with 0.52 min for the infants. The authors found that, on average, men tended to apply powder in a shorter time than women, but to use more powder and create greater airborne respirable particle concentrations [5]. 

Variability amongst subjects in the present study may be quantified using the data in Table 4. The overall mean ± SD concentration of airborne talc for the three subjects (distance = 0 cm, combining primary and secondary clouds) was 0.96 ± 0.73 mg/m^3^, indicating that variability amongst subjects was 73% RSD (RSD = SD/mean expressed as percent). This was greater than variability within any of the individual subject’s results which ranged from 15% to 46% RSD (Table 3). However, the individual subjects were applying talc using different methods, so the differences in airborne talc concentrations were likely a combination of individual behavior and the activity they were performing. High variability amongst subjects was also apparent in the results reported by Anderson et al. [6] using filter-based techniques. Considering results for the five subjects in the Anderson et al. [6] study individually, the mean ± SD values were 1.37 ± .87; 3.28 ± 1.17; 0.44 ± 0.18; 0.99 ± 0.32; and 1.15 ± 0.7, with an overall mean ± SD of 1.47 ± 1.21. Variability amongst subjects in the Anderson et al. [6] study was 83% RSD, which was greater than variability within individual results, which ranged from 32% to 63% RSD. The lower overall concentrations of respirable particle concentrations observed in the present study, compared to the Anderson et al. study, may be due in part to lower product amounts (by weight) typically used per application in the present study.

### 4.2. Additional Sources of Variability Revealed by the Chamber Studies

The real-time measurements under controlled laboratory conditions (Figure 1) facilitated a comparison with the filter-based exposure study by Anderson et al. [6], which used time-weighted averages over a 48-min measurement window. The chamber results illustrated that variability in talc cloud duration is a major source of uncertainty, as considerable “dead time” may occur between application events during the 48-min measurement window. The results of the pilot exposure study (Table 4) further confirmed that the concentration of the talc cloud strongly depends on the duration time selected for consideration, that is, higher concentrations were reported if averaged over the duration of the primary cloud only. The durations of the talc clouds in the present study were comparable to those monitored by others for similar tests: Gordon et al. [13] reported durations of approximately 55 s for their shaker test and approximately 57 s for their puff applicator test, while Russell et al. [5] reported 83 ± 33 s and Aylott et al. [4] reported 28–78 s for adult applications.

The chamber studies (Figure 1 and Table 3) also showed that the number of squeezes used to release the talc powder from the container influenced the duration of the talc cloud. A single squeeze of the baby powder container resulted in an average talc cloud duration of 7.7 min (Table 3); much longer than the average duration of 1.6 min when a triple squeeze was used to create talc clouds with the same product (Figure 1). The shorter duration of a triple squeeze talc cloud compared to a single squeeze talc cloud was attributed to disturbance of the cloud and rapid dissipation of talc particles caused by puffs of air released during subsequent squeezes of the container. This observation may relate to observations by Aylott et al. [4], who noted that the amount of accumulated respirable talc collected over four consecutive application events averaged less per event than the amount from individual application events. Another potential source of variability is the amount of talc released per squeeze of the container, which averaged between 0.23 and 0.33 g for the baby powder and body powders used in the present study, similar to that of Gordon et al. [13], who reported a weight of 0.37 g for their shaker test. Gordon et al. [13] considered this amount to be a “light application” compared to other studies: Russell et al. [5] reported applications weighing 8.84 ± 8.32 g and Aylott et al. [4] reported applications weighing 2.5 ± 12.5 g. Anderson et al. [6] reported an average mass of powder per application of 0.7 to 3.4 g.

### 4.3. Study Limitations

The small number of subjects in the pilot exposure study allowed the collection of large datasets that characterize the talc cloud generated by each subject in detail, but the results may not be representative of average exposure across the general population. The purpose of the pilot study, which employed multiple direct-reading instruments to simultaneously measure the duration, concentration and shape of the talc cloud at different distances from each subject, was to assist in the design of future exposure studies. The observed high variability of results amongst subjects warrants the inclusion of more subjects in future studies designed to assess representative population-based exposures (although the number of measurements per subject could be reduced). In addition, the subjects in the pilot study applied talc according to their own routine, rather than following a scripted application procedure. A scripted application procedure may be more appropriate for a future study designed to compare the wide variety of talc-containing products that are commercially available. Addition of a filter-based sampling component for verification of direct-reading measurements is recommended in future studies.

## 5. Conclusions

This study demonstrated that talc particles in the fine particle size range (PM_2.5_) become airborne in the PBZ during the use of talc-based cosmetics. Thus, there is a potential for inhalation exposure to insoluble silica particles during the use of cosmetic talc products, such as baby, body and face powders. The overall average concentration of airborne talc in the vicinity of the nose (combining primary and secondary clouds) was 0.96 ± 0.73 mg/m^3^ for the three subjects monitored in the present study. Individual behavior and/or different application methods strongly influenced airborne talc concentrations, demonstrated by greater variability amongst subjects (73% RSD) than within the individual subject results (15% to 46% RSD). The talc cloud characteristics varied; this could be due to varying proximity of the sampler intake to the area of talc application, different purposes of the talc application, and behavioral and physical differences amongst the subjects. Other sources of variation included the amount of air turbulence (including gusts of air from the container itself, and body movements during application), the amount of product used per application, and differences in ingredients amongst different products. 

It is concluded that the real-time, direct-reading monitoring approaches used in the present study enable detailed characterization of the short duration talc cloud that forms during product application. As direct-reading monitors can capture short-term temporal variations, they are useful for augmenting filter-based approaches which require an extended sample collection period (to exceed detection limits of gravimetric analysis). Averaging measurements over a longer time period, than the actual duration of the talc cloud, can result in a significant underestimate of airborne talc concentrations. Direct-reading measurement technologies are, therefore, recommended to assist in the evaluation of short duration inhalation exposures, required to address existing uncertainties in the evaluation of risks to talc exposure. In future, these approaches can be used to support exposure assessments of other powders in consumer products such as drywall joint compound, dry shampoo, and foot powders, in occupational as well as residential environments. 

## Figures and Tables

**Table 1:** Measurement conditions for X-ray diffraction using XRD.

Control	Setting
X-Ray (Voltage/Current)	40 kV/44 mA
Cross Beam Optics (CBO) selection slit	Bragg-Brentano focused beam
Fixed Monochromator	yes
Scan mode	continuous
Scan speed/Duration time	1.0000 degree/min.
Step width	0.0200 degree
Scan axis	2theta/theta
Scan range	20.0000–90.0000 degree
Incident slit	2/3 degree
Receiving slit #1	2/3 degree
Receiving slit #2	0.3 mm

**Table 2:** Physical-chemical characteristics of cosmetic talc products used in the present study. Particle size measurements of the talc cloud were made using the Aerodynamic Particle Sizer at a distance of 69 cm from the bottle top, inside the laboratory chamber at 27 °C and 21% RH (Relative Humidity).

Product	Ingredients (from Label)	Organic Content	Mineralogy * (XRD)	Aerodynamic Diameter (µm)	
				Median	Geomean ± Geo SD
**Baby Powder**	Talc, perfume	4.6%	≥90% talc; ≤10% chlinochlore **	2.01	2.03 ± 1.62
**Adult Body Powder #1**	Talc, zinc oxide, Zn stearate, menthol, acacia senegal gum, eucalyptol, methyl salicylate, salicylic acid, styrol	9.3%	≥98% talc	1.68	1.72 ± 1.57
**Adult Body Powder #2**	Talc, calamine powder, fragrance	1.5%	≥90% talc; ≤10% chlinochlore **	1.95	1.94 ± 1.59
**Face Powder**	Talc, ± iron oxide pigment, ± titanium oxide pigment	<3%	98% talc; 2% clinochlore **	1.95	1.88 ± 1.77

* Mineralogy expressed as percentage of inorganic content, after the organic content was removed by H_2_O_2_ digestion; ** clinochlore is a chlorite mineral (phyllosilicate group) that commonly co-occurs with talc in metamorphic rocks.

**Table 3:** Comparison of PM_10_ concentration and duration of particle cloud formed by three talc products (one-squeeze application) at varying distances from bottle top, monitored inside a laboratory chamber (*n* = 3) at 27 °C and 21% RH. See Table 2 for product characteristics.

		Baby Powder	Adult Body Powder #1	Adult Body Powder #2
30 cm ^a^	concentration (mg/m^3^)	0.87 ± 0.50	2.15 ± 0.95	0.55 ± 0.14
	time (s)	474 ± 21	520 ± 12	550 ± 22
53 cm ^b^	concentration (mg/m^3^)	5.01 ± 5.61	8.42 ± 6.71	2.06 ± 0.54
	time (s)	210 ± 53	405 ± 51	320 ± 56
69 cm ^b^	concentration (mg/m^3^)	0.39 ± 0.19	4.48 ± 6.12	0.91 ± 0.10
	time (s)	511 ± 88	602 ± 173	573 ± 37
mean ± SD	concentration (mg/m^3^)	2.09 ± 2.54	5.02 ± 3.17	1.17 ± 0.79
	time (s)	398 ± 164	509 ± 99	481 ± 140

^a^ monitored using DustTrak II; ^b^ monitored using DRX.

**Table 4:** Summary of PM_4_ concentration (mg/m^3^) and duration (s) of primary and secondary talc clouds formed during application by three subjects. Mean and standard deviation (SD) values are based on triplicate applications by each subject (secondary clouds occurred in all three replicates except where noted; dashes indicate absence of a secondary cloud). Simultaneous monitoring (at temperature 25.0–26.2 °C and RH 43–48%) of personal breathing zone at 0 cm (top) and 30 cm from face (middle), with area measurement at 53 cm from the face (bottom).

Subject	Primary		Secondary		Combined	
	mean	SD	mean	SD	mean	SD
***On Subject (Distance 0 cm)***						
***Subject A***						
Duration	57	8	-	-	-	-
Mean concentration	0.48	0.18	-	-	-	-
Max concentration	1.66	0.74	-	-	-	-
***Subject B***						
Duration	47	3	20 ^a^	7 ^a^	67	7
Mean concentration	2.48	0.83	0.43 ^a^	0.25 ^a^	1.80	0.82
Max concentration	8.33	4.07	1.39 ^a^	1.30 ^a^	8.33	4.07
***Subject C***						
Duration	192	45	508	219	700	265
Mean concentration	0.93	0.22	0.46	0.07	0.61	0.09
Max concentration	3.53	1.61	0.74	0.15	3.53	1.61
***Distance at 30 cm***						
***Subject A***						
Duration	20	8	53 ^a^	39 ^a^	73	46
Mean concentration	1.10	0.75	0.27 ^a^	0.18 ^a^	0.61	0.55
Max concentration	4.14	3.34	0.71 ^a^	0.20 ^a^	4.14	3.34
***Subject B***						
Duration	32	3	31	13	63	14
Mean concentration	0.60	0.27	0.73	0.10	0.44	0.19
Max concentration	2.78	1.39	1.00	0.22	2.78	1.39
***Subject C***						
Duration	163	37	223	58	386	93
Mean concentration	1.00	0.34	0.78	0.22	0.87	0.25
Max concentration	5.32	3.03	1.34	0.28	5.32	3.03
***Distance at 53 cm***						
***Subject A***						
Duration	70	76	59 ^b^	-	129 ^b^	-
Mean concentration	0.22	0.21	0.09 ^b^	-	0.17 ^b^	-
Max concentration	0.89	0.70	0.43 ^b^	-	0.89	0.70
***Subject B***						
Duration	199	69	-	-	-	-
Mean concentration	0.04	0.01	-	-	-	-
Max concentration	0.09	0.01	-	-	-	-
***Subject C***						
Duration	130	7	256	72	386	66
Mean concentration	0.87	0.34	0.44	0.11	0.60	0.19
Max concentration	4.38	2.30	0.75	0.14	4.38	2.30

^a^*n* = 2; ^b^ *n* = 1.

## Supplementary Materials

The following are available online at https://www.mdpi.com/1660-4601/16/20/3830/s1, Figure S1: Particle size distribution (#/cm^3^) of cosmetic talc products described in Table 2 using SMPS (<0.5 µm size range) for the baby powder and two adult body powder products, and Figure S2: Particle size distribution (#/cm^3^) of cosmetic talc products described in Table 2 using APS (≥0.5 µm size range) for all four products.
