# Supplementary material for: Characterization of Airborne Particles Emitted During Application of Cosmetic Talc Products
Source: Int J Environ Res Public Health. 2019 Oct 11;16(20):3830. doi: 10.3390/ijerph16203830 (PMC6843753; doi:10.3390/ijerph16203830)
Supplement: Supplementary file 1 [file ijerph-16-03830-s001.pdf]

International Journal of
Environmental Research and Public Health

MDPI

Supplemental Information

Characterization of airborne particles emitted during application of cosmetic talc products.

Pat E. Rasmussen [1,2*], Christine Levesque [1], Jianjun Niu [1], Howard D. Gardner [2], Gregory Nilsson [3] and Kristin Macey [4]

1 Environmental Health Science and Research Bureau, HECSB, Health Canada, 50 Colombine Drive, Ottawa, Ontario K1A 0K9, Canada.
2 Earth and Environmental Sciences Department, University of Ottawa, Ottawa, Ontario K1N 6N5, Canada.
3 Construction Research Center, National Research Council of Canada, 1200 Montreal Road, Ottawa, Ontario K1A 0R6, Canada.
4 Existing Substances Risk Assessment Bureau, HECSB, Health Canada, 269 Laurier Ave W., Ottawa, Ottawa, Ontario K1A 0P8, Canada.
* Correspondence: pat.rasmussen@canada.ca

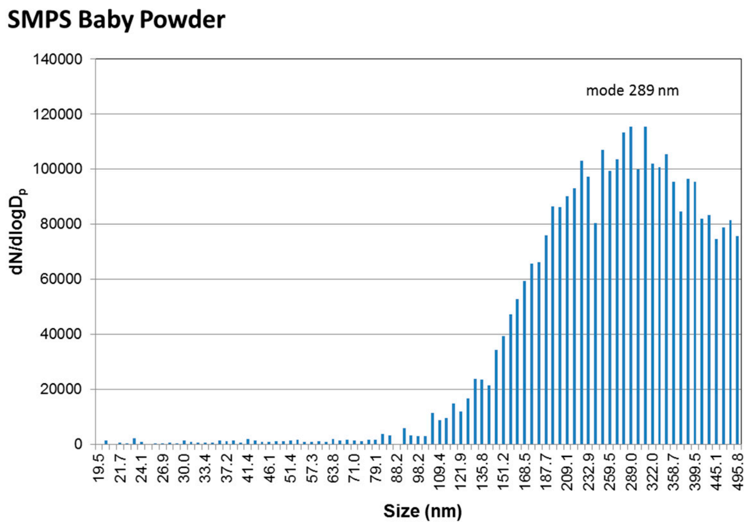

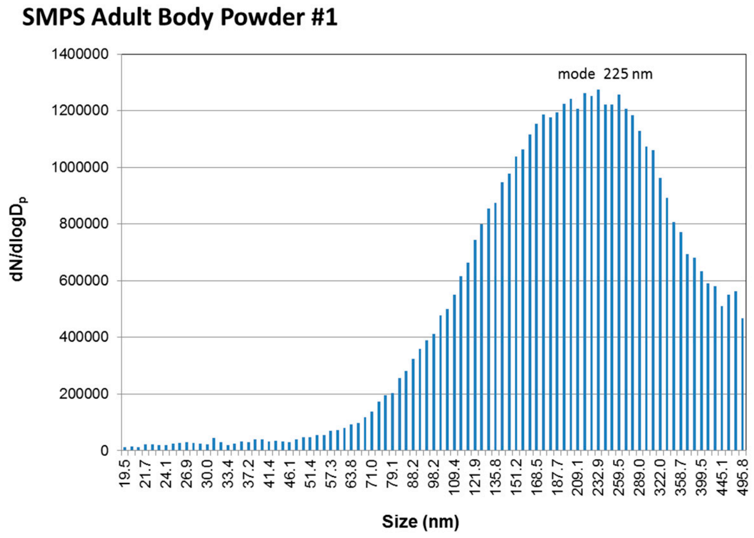

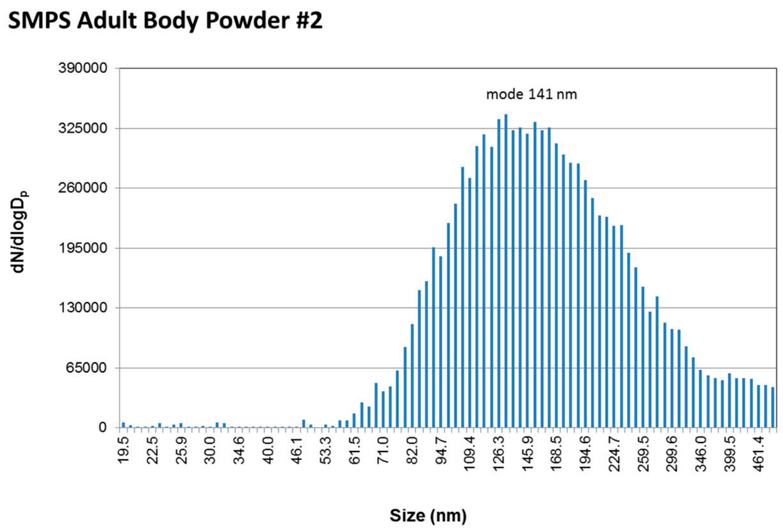

Figure S1. Particle size distribution (#/cm^3) of cosmetic talc products described in Table 2 using SMPS (<0.5 μm size range) for the baby powder and two adult body powder products.

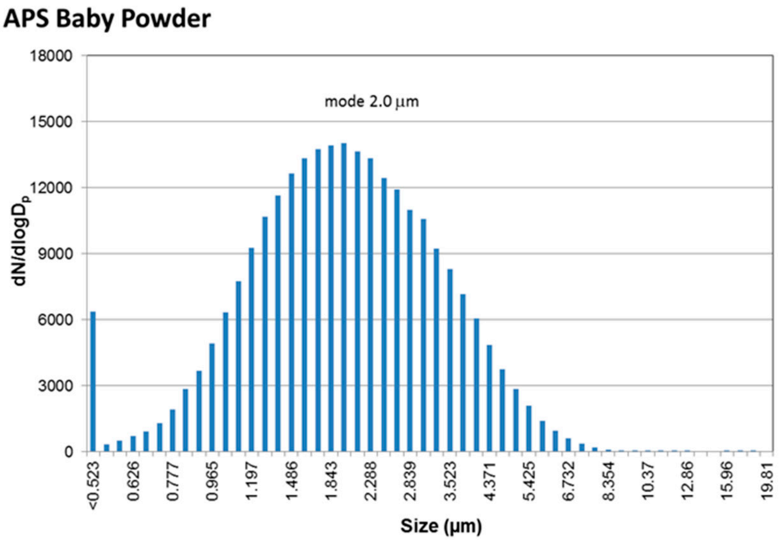

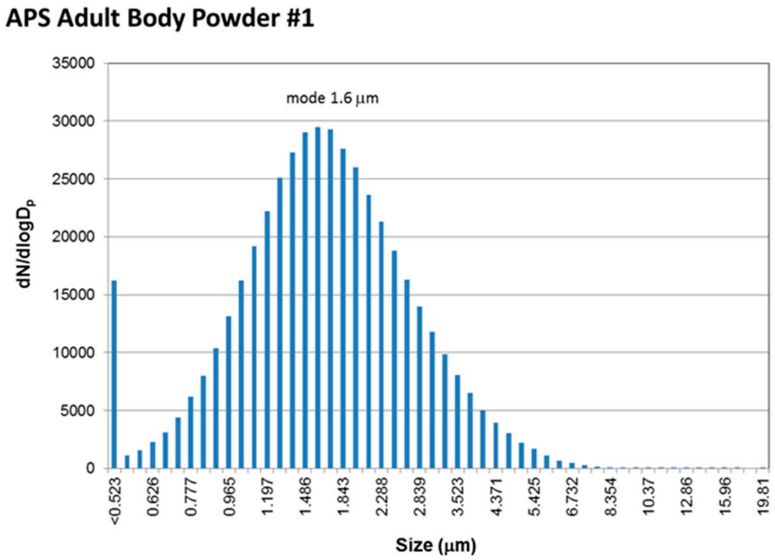

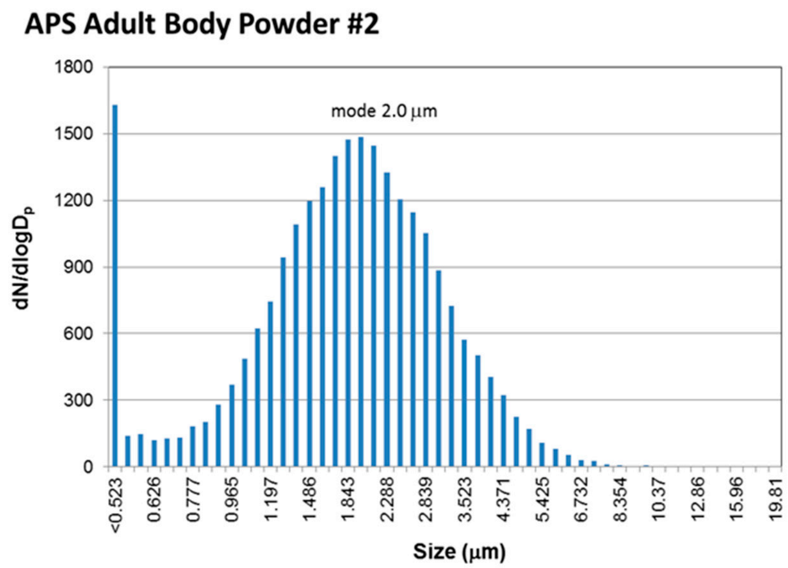

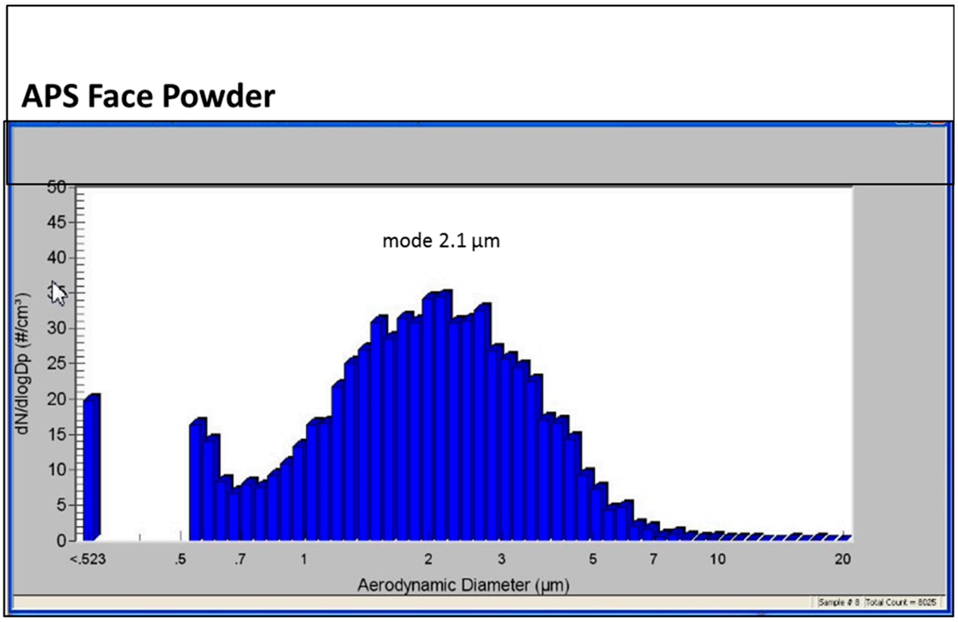

Figure S2. Particle size distribution (#/cm³) of cosmetic talc products described in Table 2 using APS (≥0.5 µm size range) for all four products.
